# Constitutive formulations for intrinsic anisotropy in soft electroelastic materials

**DOI:** 10.1038/s41598-023-37946-9

**Published:** 2023-09-07

**Authors:** Yali Li, Nakhiah C. Goulbourne

**Affiliations:** University of Michigan, Ann Arbor, USA

**Keywords:** Mechanical engineering, Polymers

## Abstract

Inspired by biology and engineered soft active material systems, we propose a new constitutive formulation for a soft material consisting of soft contractile fibers embedded in a soft matrix. The mathematical implementation of the model is based on a multi-field invariant formulation within a nonlinear continuum mechanics framework. The coupled constitutive formulation highlights a new electromechanical coupling term that describes the intrinsic (or active) anisotropy due to the contractile units. The model demonstrates the relative role that intrinsic anisotropy plays in the overall stress response. The resulting formulation could be used to design and inspire the development of new soft material systems that seek to replicate three dimensional biological motion.

## Introduction

Inspired by biology and attempts to engineer soft active material systems, we explore constitutive formulations for a soft elastic matrix containing soft contractile fibers. Although the model formulation is not tied to a particular material system, we do for now presume electric field activation to provide the external stimulus. By leveraging anisotropy, inhomogeneous deformations and complex 3D shapes can be obtained when an electric field is applied. As such, electrically-activated elastomeric materials could enable autonomous life-like motion in soft robotics for a range of applications. The constitutive model presented in this paper can be used to describe the electromechanical coupling in existing and emerging soft active and anisotropic material systems. We think the formulation is general enough that the constitutive equations proposed here could be readily particularized for increasingly complex material architectures.

### Soft fibrous materials in biology and engineering

Over the last few decades, there have been several scientific advances in developing materials and structures that mimic the functional composition and active behavior of soft biological muscle. In biology, we encounter three types of muscle cells: skeletal muscle, smooth muscle, and cardiac muscle. Smooth muscle plays an integral role in supporting contractile function in the artery via the media layer. Activity in the media layer regulates circulatory dynamics such as maintaining blood pressure and continuous blood circulation in the body. In this muscular layer, the smooth muscle cells are circumferentially oriented with a very small pitch, less than 10 degrees^1–6^. The media also contains circumferentially oriented collagen fibers, which closely envelop smooth muscle cells; a low concentration of elastin fibers are also present^2,3,7^. The heart is another interesting example of contractile function playing an integral role in cardiac dynamics. Oriented cardiac cells contract and cause a twisting motion of the heart chambers, ejecting blood into the lungs for oxygenation, and returning oxygenated blood to the rest of the body^8,9^. Skeletal muscle is usually found attached via tendons to bone for heavy purposeful force generation. Interestingly, in bat wings, the plagiopatagiales, the portion of the wing closest to the body, consists of a thin elastic membrane containing skeletal muscle fiber bundles discretely distributed and aligned in the bodylength direction^10,11^. This wing structure, unlike feathered birds, is unique and believed to enable the unique maneuverability exhibited by bats in dexterous flight^12,13^.

The development of soft contractile materials could enable many exciting applications in soft robotics due to their large deformations, light weight, low cost, and ability to achieve complex three-dimensional shapes that mimic biological motion^14–20^. Amongst the muscle-like active material candidates, liquid crystal elastomers, dielectric elastomers, McKibben actuators, and magnetic shape memory polymers have attracted the attention of the mechanics community. Of these, dielectric elastomers (DE) have demonstrated the largest actuation strains and exhibit fast electric-field actuation^21–23^. They are made by sandwiching a soft non-conducting elastomer membrane between two soft conducting layers. Applying an electric field between the conductive layers creates an electrostatic effect resulting in a thickness reduction and in plane expansion of the elastomer. In 2004, Tews et al. demonstrated an active DE membrane in a left ventricular assist device as a physiological simulator of heart pumping motion^24^. We proposed a dielectric elastomer model to describe the actuation behavior of the axisymmetrically constrained diaphragm subjected to an inflation pressure simulating blood flow^25^. Another interesting active material candidate mimicking muscle-like motions is the McKibben actuator. McKibben actuators demonstrate contractile behavior via inflation of a fiber-reinforced cylindrical bladder made from a soft elastomer^26^. In 2014, Roche et al. fabricated a composite material system consisting of discrete McKibben actuator fibers embedded in a soft elastomer^18^. They shaped the resulting composite material into a conical chamber to replicate the heart geometry and demonstrated a twisting motion when the McKibben actuators were inflated^18^.

The description of finite deformations in actively reconfigurable materials is an interesting application of continuum mechanics. Biological tissues have been modeled by the mechanics community and the applied mathematics community for several decades^27–32^. Several theories have been developed to describe and predict biological phenomena in health and disease. Much of the mechanics community has been interested in growth, remodeling, activation, and adaptation. For example, some models have been developed to describe area growth of skin, volume growth in arteries, fiber growth in the heart, and remodeling^28,29^. It is known that the mechanical quantities of stress and strain can be modulated by an electric field or other physical fields such as temperature, magnetic fields, or growth. Soft active materials like dielectric elastomers have been modeled by a stress-dependent activation inducing additional strain when a nonzero electric field is applied. We employed this approach in prior work and introduced a constitutive formulation for isotropic dielectric elastomers^25^. Insightful theoretical contributions have since addressed geometric and material instabilities^33^ by providing numerical solutions and experimental data for isotropic membranes under combined electric field and applied pressure loading. Theoretical and experimental treatments of anisotropic dielectric elastomers incorporating stiff elastic fibers have also been explored as a means for increasing the activation force and the efficiency of linear actuators^34–42^.

The idea of engineering a soft contractile material that mimics muscle is an old one. In this paper, we demonstrate an approach for modeling active anisotropy. Previous studies in shape morphing have taken the approach of adding passive fibers^36^, and patterning electrodes to spatially vary the electric field^15^: these are examples of tailoring the mechanical architecture. Recently, Davidson et al. demonstrated the ability to pattern LCE molecules in a locally varying alignment thus pre-programming compliance and realizing active compliance: this is an example of tailoring the material architecture^43^. Active anisotropy is attractive because it allows for greater control over target complex deformations by tailoring spatially varying compliance. In^43^, active anisotropy was the key for enabling more efficient dielectric liquid crystal elastomer actuators with pre-programmable actuation. These intuitive designs enabled by advanced manufacturing techniques and materials synthesis and fabrication using photoalignment methods demonstrate the potential of complex shape control via soft actuators. In spite of these experimental demonstrations, a continuum mechanics framework for studying the large deformation behavior of soft actively anisotropic materials has not been properly investigated or established.

We present a multi-field constitutive model to describe the deformation of a soft anisotropic active material that is able to contract when activated. We describe the activation mechanics in Section "Activation mechanics", and outline the continuum framework in Section "Continuum mechanics framework". We introduce a new electromechanical coupling term to capture the intrinsic activation of contractile units in the soft matrix. This means that there is coupling in the matrix and coupling in the contractile units. In Section "Results", we present simple examples to illustrate the unactivated and activated responses for various deformation states, fiber orientations, and rate parameters. Lastly, we discuss the use of the model as a tool for further studies in Section "Concluding remarks".

### Activation mechanics

**Figure 1:**
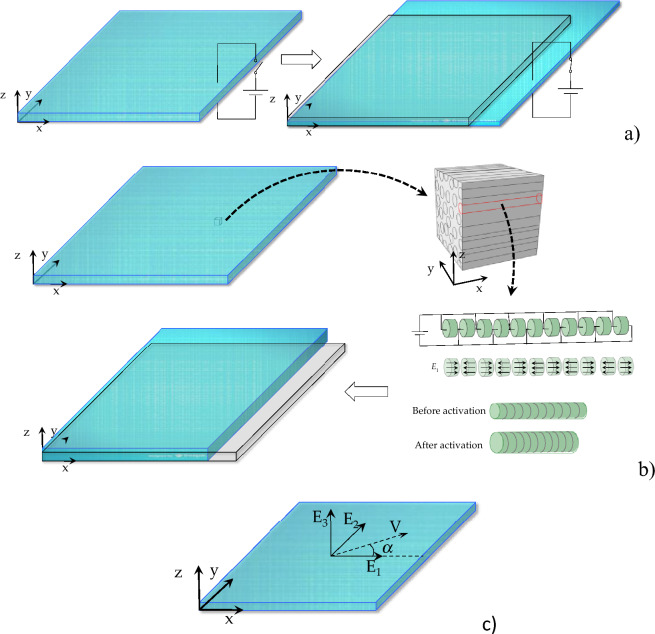
(**a**) Schematic of through-thickness matrix activation, and (**b**) schematic of fiber activation, (**c**) schematic of electric field and unit fiber orientation vectors.

Consider a soft isotropic matrix containing a family of active contractile fibers. Applying an electric field across the thickness of the matrix will result in thickness reduction and in plane membrane expansion (mode 1 activation). (Fig. 1a). In turn, the fibers contract when a component of the electric field is directed along the fiber axis (mode 2 activation). One way that the contractile units could be realized is by arranging a series of dielectric elastomer discs in a stacked configuration to form a 1D fiber (Fig. 1b) Another way would be to embed McKibben actuator fibers or consider dielectric liquid crystal elastomer actuators. Combining modes and spatial regions of actuation, distinct and unique three dimensional shapes could be attained from a single initial design.

## Continuum Mechanics Framework

Electromechanical balance equations in a thermodynamics framework together with electromechanical constitutive equations make up the system of equations to be solved. In 2005, we proposed a constitutive theory for isotropic dielectric elastomers^25^. In the same year, McMeeking and Landis presented a theoretical formulation for isotropic dielectric elastomers within a thermodynamic framework^44^. In 2008, Suo et al. provided a comprehensive and thermodynamically consistent framework for soft dielectrics, which the mechanics community has accepted for the most part^33^. Our electromechanical constitutive model has since been adopted and shown to have excellent agreement with experimental data for dielectric elastomers in a variety of actuator configurations. There have been several studies analyzing viscoelastic effects in isotropic dielectric elastomers^45–54^. The contributions of this paper are threefold: (i) to present a new constitutive model for active anisotropy in a soft material undergoing finite deformations, (ii) to account for viscoelastic effects in a thermodynamically consistent way, and (iii) to demonstrate new activation degrees of freedom through simulations. In this section, we introduce constitutive equations governing the nonlinear viscoelastic electromechanical deformation of soft actively anisotropic dielectrics.

### Kinematics

We consider a typical material point in the reference configuration ${\varvec{X}}$, where ${\varvec{\chi}}$ is the macroscopic motion that maps to a position in the current configuration. The deformation gradient is defined as

1 $${\varvec{F}}=\frac{\partial{\varvec{\chi}}}{\partial {\varvec{X}}}.$$

Viscous behavior arises both in biology and engineered polymers. In finite viscoelasticity, we apply a multiplicative decomposition of the deformation gradient into elastic and viscous parts,

2 $${\varvec{F}}={{\varvec{F}}}_{F}^{e}{{\varvec{F}}}_{F}^{v},$$

where ${{\varvec{F}}}_{F}^{e}$ and ${{\varvec{F}}}_{F}^{v}$ are elastic and viscous parts of the fiber deformation gradient,respectively. Some important strain measures that are independent of rigid body rotations are introduced here. The right Cauchy-Green deformation tensor (in the reference configuration) is defined as

3 $${\varvec{C}}={{\varvec{F}}}^{T}{\varvec{F}},\,\,\,{\varvec{C}}_{F}^{e}={{\varvec{F}}}_{F}^{eT}{{\varvec{F}}}_{F}^{e},\,\,\,{\varvec{C}}_{F}^{v}={{\varvec{F}}}_{F}^{vT}{{\varvec{F}}}_{F}^{v}.$$

Similarly, the left Cauchy-Green tensor (in the current configuration) is defined as

4 $$\mathbf{b}={\mathbf{F}\mathbf{F}}^{T}$$

#### Balance equations

From the balance of linear momentum, a spatial tensor field (Cauchy stress) ${\varvec{\sigma}}$ satisfies Cauchy’s first equation of motion in the local form (spatial description) as

5 $${div}{\varvec{\sigma}}+\mathbf{b}-\rho \dot{\mathbf{v}}=0.$$

where **b** is the body force, $\rho$ is the density, $\dot{\text{v}}$ is the acceleration, and $div$ denotes the divergence operation with respect to the current configuration. In the absence of body forces and inertial effects, Cauchy’s equation of equilibrium is obtained

6 $${div}{\varvec{\sigma}}=0.$$

In the reference configuration, Cauchy’s equation of equilibrium takes the form

7 $$DIV\mathbf{P}=0,$$

where **P** is the first Piola–Kirchhoff stress. The balance of angular momentum yields one crucial result that the Cauchy stress tensor is symmetric. The second Piola–Kirchhoff stress tensor is also symmetric, however, the first Piola-Kichhoff stress tensor is not symmetric in general. The Clausius–Duhem inequality is one strong form of the second law of thermodynamics and takes the following form in the material description,

8 $${D}_{\text{int }}=\mathbf{P}:\dot{\mathbf{F}}-\dot{\Psi }-\eta \dot{\Theta }\ge 0$$

where $\eta$ is the entropy, $\Theta$ is the absolute temperature, and $\Psi$ refers to the Helmholtz free-energy function when incorporating the thermal variables. The internal dissipation is zero for reversible processes and greater than zero for irreversible processes. Neglecting thermal effects, the inequality (8) reduces to

9 $$ D_{{\text{int }}} = \underbrace {{{\mathbf{P}}:{\dot{\mathbf{F}}}}}_{{w_{{\text{int }}} }} - {\dot{\Psi }} = w_{{\text{int }}} - {\dot{\Psi }} \ge 0, $$

where ${w}_{int}$ is the rate of internal mechanical work (or stress-power) per unit reference volume.

#### Stress tensors

Here, important forms of the stress measures are provided. The relationship between the Cauchy stress tensor and the first Piola-Kichhoff stress tensor is

10 $$\mathbf{P}=J{\varvec{\sigma}}{\mathbf{F}}^{-T}.$$

Further, the second Piola-Kichhoff stress tensor is introduced as a symmetric stress tensor and results from a complete transformation of the Cauchy stress to the reference configuration as,

11 $$\mathbf{S}={\mathbf{F}}^{-1}\mathbf{P}=J{\mathbf{F}}^{-1}{\varvec{\sigma}}{\mathbf{F}}^{-T}.$$

Even though it cannot be interpreted physically, this pure mathematical quantity is an important stress measure in computational formulations.

### Constitutive equations of activation

**Figure 2:**
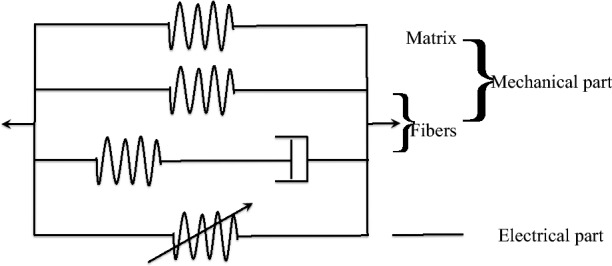
1D rheological model for a soft anisotropic electroviscoelastic material.

In finite elasticity, an invariant based formulation is commonly employed to describe a soft solid undergoing finite deformation. Soft biological materials are commonly modeled as soft fibrous continua using a similar approach. Here we have an electrically activated soft fibrous solid including viscous effects. As it might be helpful for some to visualize, we introduce a one dimensional rheological representation for infinitesimal deformation along the fiber direction in Fig. 2. The constitutive model we derive later is an extension of the rheological model to 3D finite deformations. The rheological model consists of three elements arranged in parallel: a mechanical matrix element, a mechanical fiber element, and an active element representing matrix and fiber activation. The mechanical part of the constitutive model is based on the nonlinear viscoelastic theory of solids in Reese and Govindjee^55^. Generally, the mechanical properties of the matrix can be considered isotropic and viscoelastic; here we make the simplifying assumption that the matrix is elastic. The fibers in the matrix are described by a spring representing the equilibrium response in parallel with a nonequilibruim response represented by a spring-damper for the time-dependent relaxation (Fig. 2).

Generally, for a material with two fiber families that is responsive to an electric field, we will have 12 possible invariants^56^. For a viscoelastic material with one fiber family, we introduce a strain energy function with the following functional dependence

12 $$\Psi \left({\varvec{F}}, {{\varvec{F}}}_{F}^{v},{\varvec{M}},{\varvec{E}}\right)=\overline{\Psi  }\left({I}_{1}\sim {I}_{4},{\mathrm{I}}_{1}^{v}\sim {\mathrm{I}}_{4}^{v},{I}_{10}\sim {I}_{12}\right),$$

where ***M*** is the so-called structural tensor accounting for anisotropic behavior, ***E*** is the nominal electric field, and, I_i_ are the principal invariants. We ignore any potential dependence on cross-coupling terms and higher order terms for simplicity. We also renumber the invariants for continuity and clarity in the following.

For an isotropic material, the principal invariants are expressed in terms of the right Cauchy-Green tensor as

13 $${I}_{1}={tr}\left[\mathbf{C}\right],{I}_{2}={tr}\left[{cof}\left[\mathbf{C}\right]\right],{I}_{3}={det}\left[\mathbf{C}\right],$$

where ${I}_{1},{I}_{2},{I}_{3}$ represent the length, area, and volume change of the solid; *tr*, *cof*, *det* are the trace, cofactor, and determinant of the right Cauchy-Green tensor, respectively. In the current configuration, we have **v** = **FV**. Figure 1c shows an illustration of the fiber orientation vector for a planar distribution and the electric field vector. Following Spencer^57–61^, the principal invariant for the contractile fibers in the composite matrix are

14 $${I}_{4}=\mathbf{V}\cdot\mathbf{C}\mathbf{V}=\mathbf{C}:\mathbf{M},$$

where ${I}_{4}$ is the square of the fiber stretch. **M** is the structure tensor and ${\varvec{M}} = {\mathbf{V}} \otimes {\mathbf{V}}$. Fiber orientation vector and structure tensor in the current configuration are $\mathbf{v}={\varvec{F}}\mathbf{V}$ and ${\mathbf{m}} = {\mathbf{v}} \otimes {\mathbf{v}}$. The elastic part of the fourth stretch invariant ${I}_{4}^{e}$ is defined as ${\left({\lambda }_{F}^{e}\right)}^{2}$. The relationship between the viscous and elastic parts is

15 $$ \left( {\lambda_{F}^{e} } \right)^{2} = \frac{{I_{4} }}{{I_{4}^{v} }} = {\mathbf{V}}\text{*} \cdot {\mathbf{C}}_{{\varvec{F}}}^{{\varvec{e}}} {\mathbf{V}}\text{*} = \frac{{{\mathbf{V}} \cdot {\mathbf{CV}}}}{{{\mathbf{V}} \cdot {\mathbf{C}}_{{\varvec{F}}}^{{\varvec{v}}} {\mathbf{V}}}} = \lambda_{F}^{e} {\text{v}^{\prime}} $$

where V* is the unit fiber orientation in the intermediate configuration and ${\varvec{V}}\text{*}={\mathbf{F}}_{F}^{v}\mathbf{V}/\Vert {\mathbf{F}}_{F}^{v}\mathbf{V}\Vert$.

The normalized fiber orientation vector in the current configuration is

16 $$ {\mathbf{v^{\prime}}} = \frac{{\mathbf{v}}}{{\left| {\mathbf{v}} \right|}} = \frac{{\mathbf{v}}}{{({\mathbf{v}} \cdot {\mathbf{v}})^{1/2} }} = \frac{{\mathbf{v}}}{{\left( {I_{4} } \right)^{1/2} }} = \frac{{\mathbf{v}}}{\lambda }. $$

where $\lambda$ is the fiber stretch.

The principal invariants associated with the electric field^56^, are defined as

17 $$ I_{5} = {\mathbf{E}} \cdot {\mathbf{E}},I_{6} = {\mathbf{E}} \cdot {\mathbf{C}}^{ - 1} \cdot {\mathbf{E}}{. } $$

The invariants ${I}_{5}$ and ${I}_{6}$ represent the quadratics of the nominal and true electric field **E** and **e**. Consider components of the applied electric field and the unit fiber orientation vector given by

18 $$ {\mathbf{E}} = \left[ {\begin{array}{*{20}c} {E_{1} } & {E_{2} } & {E_{3} } \\ \end{array} } \right]^{T} { }\;\;{\text{and}}\;\;{\mathbf{V}} = \left[ {\begin{array}{*{20}c} {{\text{cos}}\alpha } & {{\text{sin}}\alpha } & 0 \\ \end{array} } \right]^{T} . $$

Assuming it is experimentally feasible, we imagine activating the fibers along their axis as

19 $${E}_{f}=\mathbf{E}\cdot \mathbf{V}={E}_{1}\mathrm{cos}\alpha +{E}_{2}\mathrm{sin}\alpha .$$

This is the magnitude of the nominal electric field in the fiber direction. Therefore, we write the electric field activation of the matrix and of the fibers as

20 $$ {\mathbf{E}}_{m} = \left[ {\begin{array}{*{20}c} 0 & 0 & {E_{3} } \\ \end{array} } \right]^{T} ,\;\;\;\;E_{f} = E_{1} {\text{cos}}\alpha + E_{2} \sin \alpha , $$

and rewrite ${I}_{6}$ in terms of matrix and fiber parts

21 $${I}_{6}^{m}={\mathbf{E}}_{m}\cdot {\mathbf{C}}^{-1}\cdot {\mathbf{E}}_{m},{I}_{6}^{f}={\left(\frac{{E}_{f}}{\lambda }\right)}^{2}=\frac{{E}_{f}^{2}}{{I}_{4}},$$

where $\lambda$ is the fiber stretch and therefore ${E}_{f}/\lambda$ is the true electric field in the fiber.

### Explicit Functional Forms of the Stress: Electromechanical coupling

The previous section presented generalized functional forms of the constitutive equations for soft fibrous active continua. In this section, we assume incompressibility and introduce the invariant-based strain energy function as an additive combination of purely mechanical and electromechanical contributions. The mechanical part captures the elastic and viscous behavior and the electromechanical part captures the coupling response. For simplicity, we employ a neo-Hookean expression to describe the elastic response of the matrix i.e. dependence on the first elastic invariant where the linear coefficient $\mu$ is the shear modulus of the matrix. The mechanical part of the fiber response is split into equilibrium and nonequilibrium parts to capture the viscoelastic behavior. The equilibrium term presumes dependence on ${I}_{4}$, which is the square of the fiber stretch, and the proportionality constant ${\mu }_{feq}$ is the equilibrium part of the fiber modulus. The nonequilibrium fiber response has a presumed quadratic dependence on ${I}_{4}/{I}_{4v}$ and ${\mu }_{fneq}$ is then the nonequilibrium part of the fiber modulus. The passive part of the formulation is identical in form to those used for modeling viscoelasticity in soft biological tissues. For the electromechanical coupling in the matrix, we employ a quadratic dependence on the true electric field by introducing a term that is linearly proportional to ${I}_{6}$, where we use the definitions in Eq. (21). This linear coefficient ${\varepsilon }_{rm}$ is the dielectric constant. We introduce a similar expression for the electromechanical coupling in the fiber, and by analogy identify the proportionality constant ${\varepsilon }_{rf}$ as the fiber dielectric constant. The strain energy formulation, grouped into purely mechanical parts and electromechanical parts, is then given as

22 $$ {\Psi } = \underbrace {{\frac{\mu }{2}\left( {I_{1} - 3} \right) + \frac{{\mu_{{{feq }}} }}{2}\left( {I_{4} - 1} \right)^{2} + \frac{{\mu_{{{freq }}} }}{2}\left( {\frac{{I_{4} }}{{I_{4}^{v} }} - 1} \right)^{2} }}_{mechanical }\underbrace {{ - \frac{{\varepsilon_{0} \varepsilon_{rf} }}{2}I_{6}^{f} }}_{electrical\text{-}fiber }\underbrace {{ - \frac{{\varepsilon_{0} \varepsilon_{rm} }}{2}I_{6}^{m} }}_{electrical\text{-}matrix}, $$

where ${\varepsilon }_{0}$ is the vacuum permittivity. The Second Piola–Kirchhoff stress is derived from the Clausis-Duhem inequality by performing the following operation

23 $$\mathbf{S}=2\frac{\partial\Psi }{\partial \mathbf{C}}.$$

It follows that

24 $$ \begin{aligned} {\mathbf{S}} & = \underbrace {{\mu {\mathbf{I}} + 2\mu_{{{feq }}} \left( {I_{4} - 1} \right){\mathbf{M}} + 2\mu_{{{fneq }}} \left( {\frac{{I_{4} }}{{I_{4}^{v} }} - 1} \right)\frac{{\mathbf{M}}}{{I_{4}^{v} }}}}_{mechanical } + \underbrace {{\frac{{\varepsilon_{0} \varepsilon_{rf} }}{{I_{4}^{2} }}E_{f}^{2} {\mathbf{M}}}}_{electrical\text{-}fiber } \\ & \quad + \underbrace {{\frac{{\varepsilon_{0} \varepsilon_{rm} }}{2}\left( {{\mathbf{C}}^{ - 1} \odot {\mathbf{C}}^{ - 1} } \right):\left( {{\mathbf{E}}_{m} \otimes {\mathbf{E}}_{m} } \right)}}_{electrical\text{-}matrix} \\ \end{aligned} $$

where we use the relations

25 $$\frac{\partial {\mathbf{C}}^{-1}}{\partial \mathbf{C}}=-{\mathbf{C}}^{-1}\odot {\mathbf{C}}^{-1}$$

and

26 $$\partial {C}_{AB}^{-1}/\partial {C}_{CD}=-1/2\left({C}_{AC}^{-1}{C}_{BD}^{-1}+{C}_{AD}^{-1}{C}_{BC}^{-1}\right).$$

The Cauchy stress is thus derived using the operation

27 $${\varvec{\sigma}}={\mathbf{F}\mathbf{S}\mathbf{F}}^{T}+p\mathbf{I}.$$

where *-p* is the hydrostatic pressure. Grouping the stress into three parts: mechanical, electrical-fiber, and electrical-matrix, the total Cauchy stress is then derived as

28 $$ \begin{aligned} {\varvec{\sigma}} & = \underbrace {{\mu {\mathbf{b}} + 2\mu_{{{feq }}} \left( {I_{4} - 1} \right){\mathbf{v}} \otimes {\mathbf{v}} + 2\mu_{{{freq }}} \left( {\frac{{I_{4} }}{{I_{4}^{v} }} - 1} \right)\frac{1}{{I_{4}^{v} }}{\mathbf{v}} \otimes {\mathbf{v}}}}_{mechanical } \\ & \quad + \underbrace {{\varepsilon_{0} \varepsilon_{rf} \frac{{E_{f}^{2} }}{{I_{4} }}\frac{{\mathbf{v}}}{{\sqrt {I_{4} } }} \otimes \frac{{\mathbf{v}}}{{\sqrt {I_{4} } }}}}_{electrical\text{-}fiber } + \underbrace {{\varepsilon_{0} \varepsilon_{rm} {\mathbf{e}}_{m} \otimes {\mathbf{e}}_{m} - \frac{{\varepsilon_{0} \varepsilon_{rm} }}{2}{\mathbf{e}}_{m} \cdot {\mathbf{e}}_{m} {\mathbf{I}}}}_{{{electrical\text{-}matrix }}} + p{\mathbf{I}}, \\ \end{aligned} $$

Recall that the true electric field for the fiber part is ${{E}_{f}}/{\lambda }$ , the true electric field for the isotropic matrix is ${{\varvec{e}}}_{m}={{\varvec{F}}}^{-T}{{\varvec{E}}}_{m}$, and ${\text{v}}/{\sqrt{{I}_{4}}}$ is the unit fiber vector in the current configuration. The three terms in the mechanical part describe the elastic behavior of the matrix and the viscoelastic behavior of the fibers. If there is no applied electric field, then only the mechanical part remains. The mechanical anisotropy depends on the ratio between the matrix term and fiber term (orientation). For an electric field in the matrix only, the electromechanical fiber coupling term drops out and we recover the familiar Maxwell stress tensor in the electrical part of the stress

29 $$ \begin{aligned} {\varvec{\sigma}} & = \underbrace {{\mu {\mathbf{b}} + 2\mu_{{feq }} \left( {I_{4} - 1} \right){\mathbf{v}} \otimes {\mathbf{v}} + 2\mu_{{fneq }} \left( {\frac{{I_{4} }}{{I_{4}^{v} }} - 1} \right)\frac{1}{{I_{4}^{v} }}{\mathbf{v}} \otimes {\mathbf{v}}}}_{mechanical } \\ & \quad + \underbrace {{\varepsilon_{0} \varepsilon_{rm} {\mathbf{e}}_{m} \otimes {\mathbf{e}}_{m} - \frac{{\varepsilon_{0} \varepsilon_{rm} }}{2}{\mathbf{e}}_{m} \cdot {\mathbf{e}}_{m} {\mathbf{I}}}}_{Maxwell\,\, stress\,\, tensor } + p{\mathbf{I}}, \\ \end{aligned} $$

In the absence of embedded fibers, the stress in Eq. (28) is reduced to a small strain constitutive model for isotropic dielectric elastomers

30 $$ {\varvec{\sigma}} = \underbrace {{\mu {\mathbf{b}}}}_{mechanical } + \underbrace {{\varepsilon_{0} \varepsilon_{rm} {\mathbf{e}}_{m} \otimes {\mathbf{e}}_{m} - \frac{{\varepsilon_{0} \varepsilon_{rm} }}{2}{\mathbf{e}}_{m} \cdot {\mathbf{e}}_{m} {\mathbf{I}}}}_{Maxwell \,\,stress\,\, tensor } + p{\mathbf{I}}. $$

The mechanical part of the stress formulation in Eq. (28) could be replaced with the Mooney-Rivlin model, Yeoh’s model, and Gent model etc. as follows to capture the initial modulus and strain softening behavior of elastomeric matrices

31 $$ \begin{aligned} {\varvec{\sigma}} & = \underbrace {{{\varvec{\mu}}\left( {{\mathbf{b}} + {\varvec{\gamma}}{\mathbf{b}}^{ - 1} } \right)}}_{Mooney\text{-}Rivlin } + \underbrace {{2{\varvec{\mu}}_{{\text{feq }}} \left( {{\varvec{I}}_{4} - 1} \right){\mathbf{v}} \otimes {\mathbf{v}} + 2{\varvec{\mu}}_{{\text{fneq }}} \left( {\frac{{{\varvec{I}}_{4} }}{{{\varvec{I}}_{4}^{{\varvec{v}}} }} - 1} \right)\frac{1}{{{\varvec{I}}_{4}^{{\varvec{v}}} }}{\mathbf{v}} \otimes {\mathbf{v}}}}_{mechanical } \\ & \quad + \underbrace {{{\varvec{\varepsilon}}_{0} {\varvec{\varepsilon}}_{{{\varvec{rf}}}} \frac{{{\varvec{E}}_{{\varvec{f}}}^{2} }}{{{\varvec{I}}_{4} }}\frac{{\mathbf{v}}}{{\sqrt {{\varvec{I}}_{4} } }} \otimes \frac{{\mathbf{v}}}{{\sqrt {{\varvec{I}}_{4} } }}}}_{electrical\text{-}fiber } + \underbrace {{{\varvec{\varepsilon}}_{0} {\varvec{\varepsilon}}_{{{\varvec{rm}}}} {\mathbf{e}}_{{\varvec{m}}} \otimes {\mathbf{e}}_{{\varvec{m}}} - \frac{{{\varvec{\varepsilon}}_{0} {\varvec{\varepsilon}}_{{{\varvec{rm}}}} }}{2}{\mathbf{e}}_{{\varvec{m}}} \cdot {\mathbf{e}}_{{\varvec{m}}} {\mathbf{I}}}}_{electrical\text{-}matrix } + {\varvec{p}}{\mathbf{I}}, \\ \end{aligned} $$

32 $$ \begin{aligned} {\varvec{\sigma}} & = \underbrace {{\underbrace {{\sum\limits_{{{\varvec{i}} = 1}}^{{\varvec{n}}} {\varvec{i\mu }_{{\varvec{i}}} \left( {{\varvec{I}}_{1} - 3} \right)^{{{\varvec{i}} - 1}} {\mathbf{b}}} }}_\text{Yeoh's model } + 2{\varvec{\mu}}_{{ {feq }}} \left( {{\varvec{I}}_{4} - 1} \right){\mathbf{v}} \otimes {\mathbf{v}} + 2{\varvec{\mu}}_{{ {fneq }}} \left( {\frac{{{\varvec{I}}_{4} }}{{{\varvec{I}}_{4}^{{\varvec{v}}} }} - 1} \right)\frac{1}{{{\varvec{I}}_{4}^{{\varvec{v}}} }}{\mathbf{v}} \otimes {\mathbf{v}}}}_{mechanical} + \underbrace {{{\varvec{\varepsilon}}_{0} {\varvec{\varepsilon}}_{{{\varvec{rf}}}} \frac{{{\varvec{E}}_{{\varvec{f}}}^{2} }}{{{\varvec{I}}_{4} }}\frac{{\mathbf{v}}}{{\sqrt {{\varvec{I}}_{4} } }} \otimes \frac{{\mathbf{v}}}{{\sqrt {{\varvec{I}}_{4} } }}}}_{electrical\text{-}fiber } \\ &\quad + \underbrace {{{\varvec{\varepsilon}}_{0} {\varvec{\varepsilon}}_{{{\varvec{rm}}}} {\mathbf{e}}_{{\varvec{m}}} \otimes {\mathbf{e}}_{{\varvec{m}}} - \frac{{{\varvec{\varepsilon}}_{0} {\varvec{\varepsilon}}_{{{\varvec{rm}}}} }}{2}{\mathbf{e}}_{{\varvec{m}}} \cdot {\mathbf{e}}_{{\varvec{m}}} {\mathbf{I}}}}_{electrical\text{-}matrix } + {\varvec{p}}{\mathbf{I}}, \\ \end{aligned} $$

33 $$ \begin{aligned} {\varvec{\sigma}} & = \underbrace {{\underbrace {{\frac{{\user2{\mu J}_{{\varvec{m}}} }}{{{\varvec{J}}_{{\varvec{m}}} - {\varvec{I}}_{1} + 3}}{\mathbf{b}}}}_{{{\text{Gent}}\;{\text{model}}}} + 2{\varvec{\mu}}_{feq } \left( {{\varvec{I}}_{4} - 1} \right){\mathbf{v}} \otimes {\mathbf{v}} + 2{\varvec{\mu}}_{fneq } \left( {\frac{{{\varvec{I}}_{4} }}{{{\varvec{I}}_{4}^{{\varvec{v}}} }} - 1} \right)\frac{1}{{{\varvec{I}}_{4}^{{\varvec{v}}} }}{\mathbf{v}} \otimes {\mathbf{v}}}}_{mechanical} \\ &\quad + \underbrace {{{\varvec{\varepsilon}}_{0} {\varvec{\varepsilon}}_{{{\varvec{rf}}}} \frac{{{\varvec{E}}_{{\varvec{f}}}^{2} }}{{{\varvec{I}}_{4} }}\frac{{\mathbf{v}}}{{\sqrt {{\varvec{I}}_{4} } }} \otimes \frac{{\mathbf{v}}}{{\sqrt {{\varvec{I}}_{4} } }}}}_{electrical\text{-}fiber} + \underbrace {{{\varvec{\varepsilon}}_{0} {\varvec{\varepsilon}}_{{{\varvec{rm}}}} {\mathbf{e}}_{{\varvec{m}}} \otimes {\mathbf{e}}_{{\varvec{m}}} - \frac{{{\varvec{\varepsilon}}_{0} {\varvec{\varepsilon}}_{{{\varvec{rm}}}} }}{2}{\mathbf{e}}_{{\varvec{m}}} \cdot {\mathbf{e}}_{{\varvec{m}}} {\mathbf{I}}}}_{electrical\text{-}matrix} + {\varvec{p}}{\mathbf{I}}, \\ \end{aligned} $$

where ${\varvec{\gamma}},{\varvec{n}},{{\varvec{J}}}_{{\varvec{m}}}$ are model parameters.

#### Evolution equation

We consider rate effects in the fibers that might arise due to internal energy storing and dissipating mechanisms in the active contractile units. In finite inelasticity, this means that the evolution equation governing the internal variable ${I}_{4}^{v}$ needs to satisfy the Clausius–Duhem inequality derived from the second law of thermodynamics. Here, we employ the following linear form^55^

34 $$-{\tau }_{\text{neq }}=\eta {\dot{I}}_{4}^{v},$$

35 $${\tau }_{{neq }}=4{I}_{4}^{v}\frac{\partial\Psi }{\partial {I}_{4}^{v}}=4{\mu }_{{fneq }}\left(\frac{{I}_{4}}{{I}_{4}^{v}}-1\right)\frac{{I}_{4}}{{I}_{4}^{v}}.$$

This form has been widely adopted to model viscous effects in soft biological materials such as muscle. These expressions round out the model formulation together with Eqs. (22) and (28).

## Results

We consider the finite deformation behavior of a soft fibrous actively contractile material using the newly developed constitutive formulation. We study the relative contributions of the terms in the constitutive model for two simple cases: uniaxial extension and equibiaxial extension. The results show that the interplay of matrix and fiber coupling can amplify or mute the coupled electromechanical response.

### Uniaxial extension

We consider a family of in-plane fibers aligned in the x-direction and apply a uniaxial deformation in the x-direction. The unit fiber orientation vector and the deformation gradient are then

$$\mathbf{V}={\left[\begin{array}{ccc}1& 0& 0\end{array}\right]}^{T}\,\,\,\,\mathbf{F}=\mathrm{diag}\left[\begin{array}{ccc}\lambda & {\lambda }^{-\frac{1}{2}}& {\lambda }^{-\frac{1}{2}}\end{array}\right].$$

We apply an electric field along the fiber direction, thus

$$\mathbf{E}={\left[\begin{array}{ccc}E& 0& 0\end{array}\right]}^{T}.$$

The left Cauchy Green tensor is

$$\mathbf{b}=\mathrm{diag}\left[\begin{array}{ccc}{\lambda }^{2}& {\lambda }^{-1}& {\lambda }^{-1}\end{array}\right]$$

and the fiber vector in the current configuration is

$$\mathbf{v}=\mathbf{F}\mathbf{V}={\left[\begin{array}{ccc}\lambda & 0& 0\end{array}\right]}^{T}.$$

The stress in the x-direction is obtained from Eq. (28). Using the following boundary conditions

$${\sigma }_{22}={\sigma }_{33}=0,$$

We have

36 $${\sigma }_{11}=\mu \left({\lambda }^{2}-\frac{1}{\lambda }\right)+2{\mu }_{\text{feq }}\left({\lambda }^{2}-1\right){\lambda }^{2}+2{\mu }_{fneq}\left(\frac{{\lambda }^{2}}{{I}_{4}^{v}}-1\right)\frac{{\lambda }^{2}}{{I}_{4}^{v}}+{\varepsilon }_{0}{\varepsilon }_{rf}\frac{{E}^{2}}{{\lambda }^{2}}.$$

The nondimensional stress (denoted by an overbar) is obtained by dividing this expression by the matrix shear modulus

37 $$ \overline{\sigma } = \left( {\lambda^{2} - \frac{1}{\lambda }} \right) + 2\overline{\mu }_{feq} \left( {\lambda^{2} - 1} \right)\lambda^{2} + 2\overline{\mu }_{fneq } \left( {\frac{{\lambda^{2} }}{{I_{4}^{v} }} - 1} \right)\frac{{\lambda^{2} }}{{I_{4}^{v} }} + \frac{{\overline{E}^{2} }}{{\lambda^{2} }}. $$

The relationship between the dimensional and nondimensional variables are

38 $$ \overline{\sigma }_{11} = \frac{{\sigma_{11} }}{\mu },\;\overline{\mu }_{feq } = \frac{{\mu_{feq } }}{\mu },\overline{\mu }_{freq } = \frac{{\mu_{fneq } }}{\mu }{, }\;\;{\text{and }}\;\;\overline{E} = \left( {\frac{{\varepsilon_{0} \varepsilon_{rf} }}{\mu }} \right)^{1/2} E, \;\overline{\eta } = \frac{\eta }{{4\mu_{fneq } }}. $$

The nondimensional evolution equation is obtained by dividing the nonequliubrium fiber shear modulus ${\mu }_{fneq}$ (which yields the characteristic relaxation time) as

39 $$\left(\frac{{\lambda }^{2}}{{I}_{4}^{v}}-1\right)\frac{{\lambda }^{2}}{{I}_{4}^{v}}=\overline{\eta }{\dot{I}}_{4}^{v}.$$

**Figure 3:**
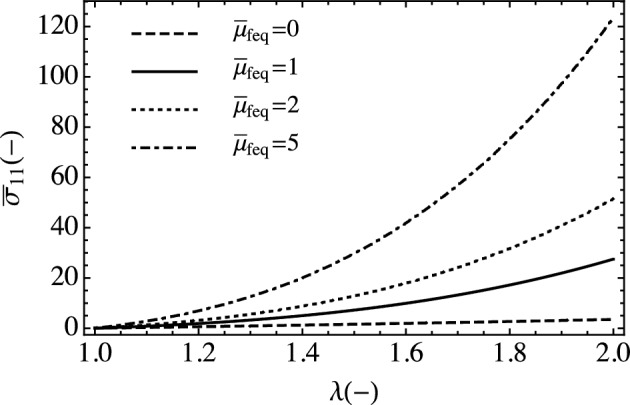
Uniaxial plots of the passive stress term with different fiber:matrix modulus ratios.

For simple stress states, we determine the relative importance of the material modulus, characteristic time, dielectric constant, and fiber orientation in the model. We set the electric field to zero to focus on the mechanical part of the constitutive model for a range of fiber moduli values. Figure 3 shows the stress stretch curves for quasi-static loading ($\dot{\lambda }=0.001 /s$) where it is seen that they are qualitatively similar to Fung-type and structure-informed models of soft biological tissue.

**Figure 4:**
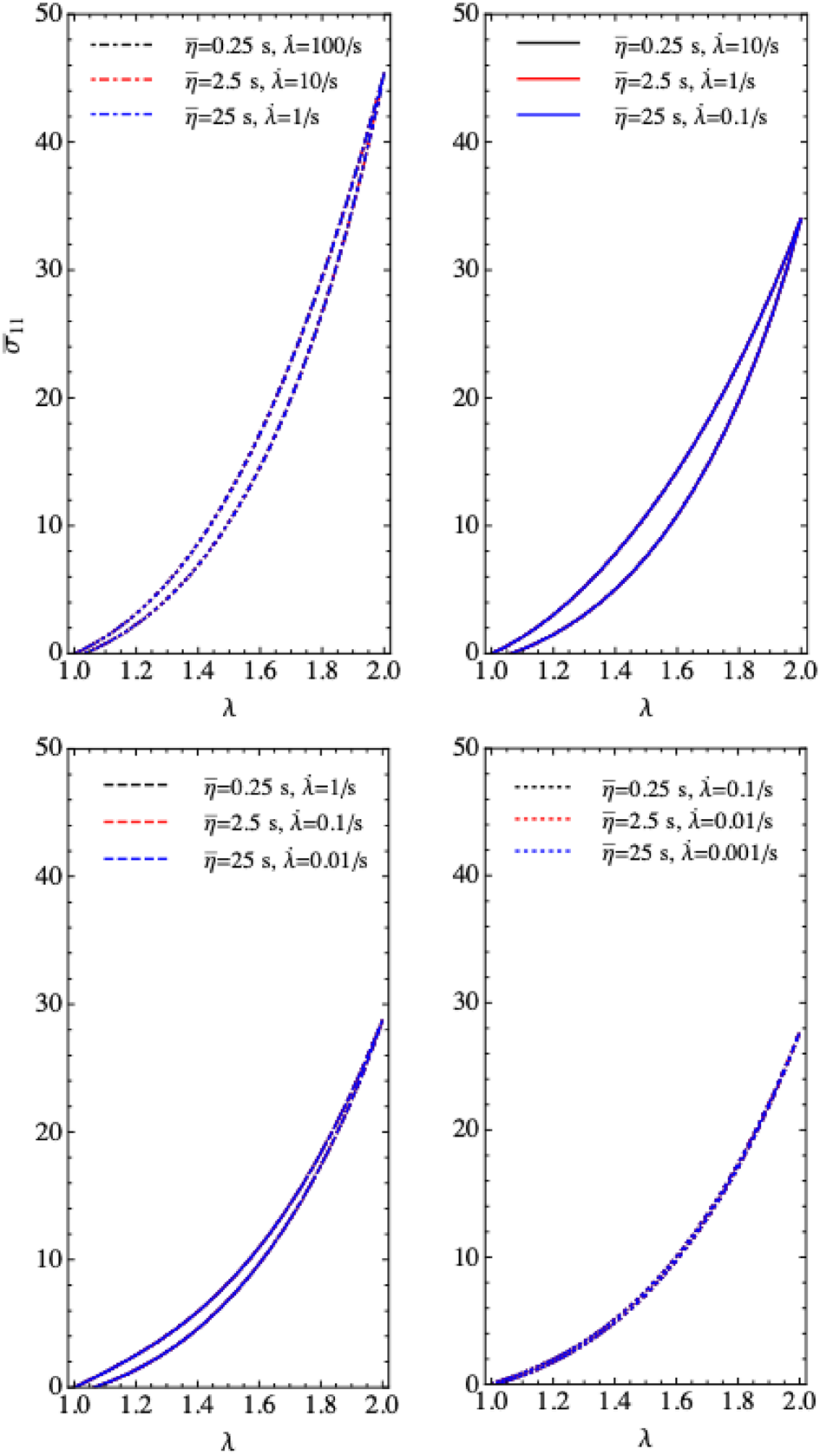
Uniaxial plots of the viscoelastic terms with different loading rates.

Next, we consider the effect of the viscous parameter on the passive response for various loading/unloading rates. Figure 4 shows the nondimensional uniaxial stress for a range of viscous parameters $\overline{\eta }$ (characteristic relaxation times). The sample is stretched to twice its initial length with various stretch rates (unloaded with the same rates). The model captures stiffening effects and rate-dependent energy dissipation: larger stresses are generated for faster loadings, and the hysteresis loops disappear for quasi-static loading. It shows that for the same $\overline{\eta }\cdot \dot{\lambda }$, the stress stretch curves are the same.

**Figure 5:**
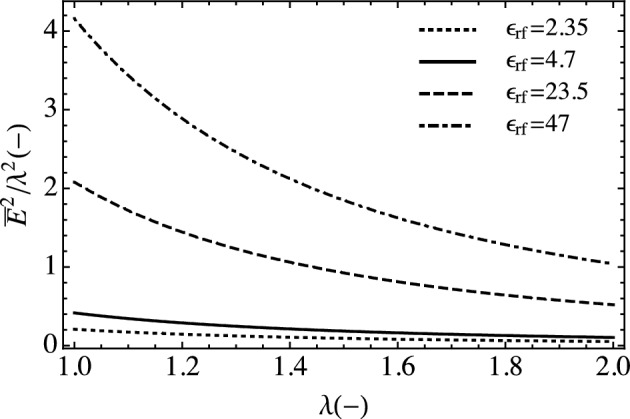
Plot of the nondimensional uniaxial electromechanical coupling term with different dielectric constants.

Now we look at the electromechanical coupling term in the constitutive equation. Figure 5 illustrates the functional dependence of the activation stress on fiber dielectric constant as we stretch the material. The selected values are informed by existing materials. Typical dielectric elastomers have dielectric constants between 2 and 5 whereas the dielectric constant for LCEs is much higher owing to their local polarization. The electromechanical coupling term will dominate the response in the initial stretch regime, having less impact at larger stretch.

**Figure 6:**
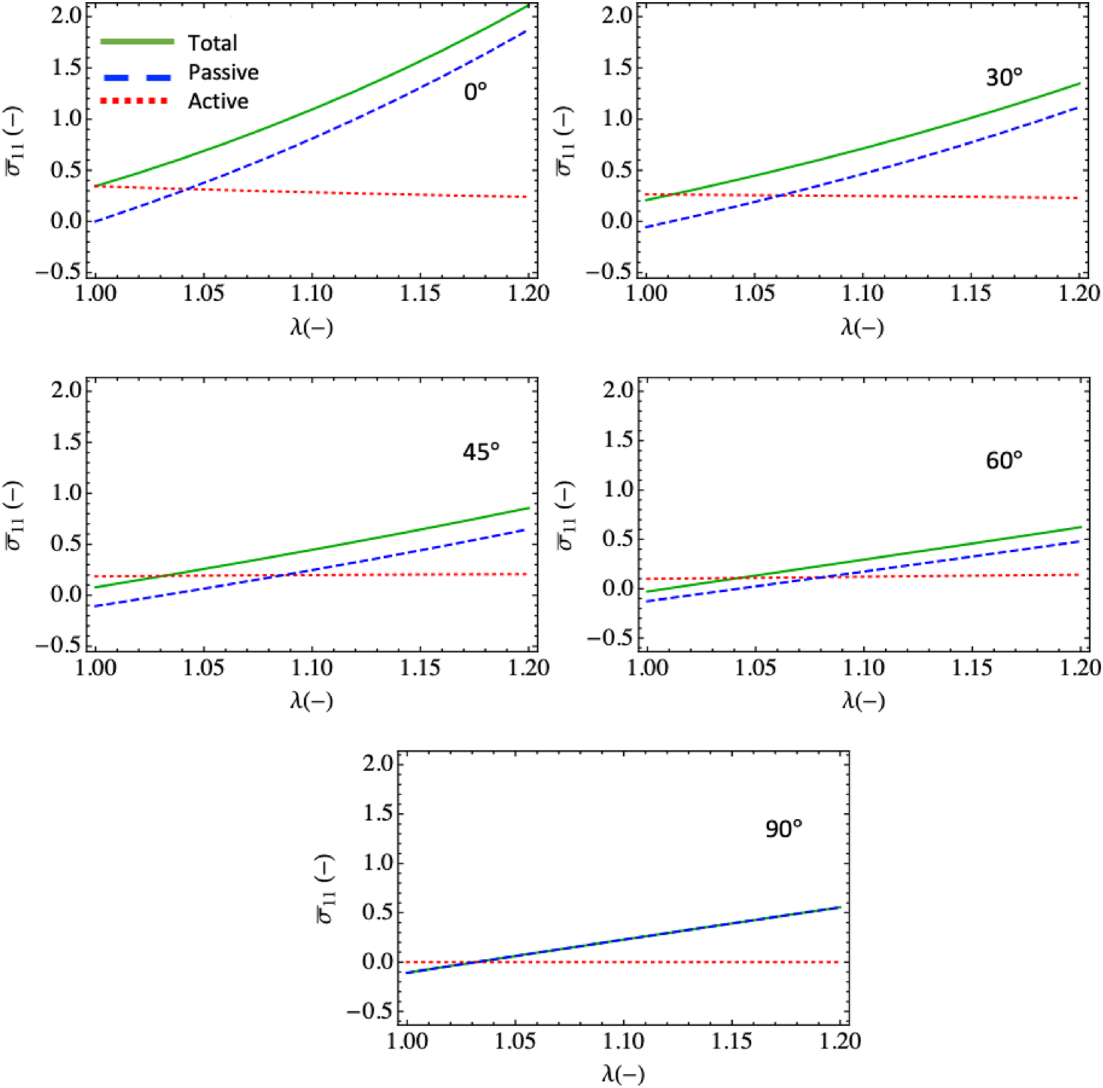
Passive, active, and total uniaxial stress with fiber orientations 0°, 30°, 45°, 60°, 90° (left to right).

To show the relative contributions of the purely mechanical and electromechanical parts in the constitutive response we calculate the total, active, and passive stress for varying fiber orientations (0:90 degrees with respect to x). We use a modest applied electric field of 12 MV/m along the fibers. The results show that the active contribution is relatively small and uniform for the selected material parameters and stretch range considered (Fig. 6). Increasing the magnitude of the coupling term (dielectric constant or electric field) will increase the offset. In the results, we also see that the largest electromechanical response is obtained when the fibers are in full alignment with the applied field. The active stress decreases with increasing misalignment. This is to be expected as the coupling occurs only for the component of the fiber orientation that is projected onto the field direction. That is to say, there is full coupling when the fibers are oriented at 0° and no coupling when fibers are at 90°. The results illustrate the interplay and relative influence of the active and passive terms in the constitutive response. The active term creates an initial offset and the passive term dominates with increasing stretch. The fiber term dominates when fibers are aligned and the matrix term dominates when the fibers are not aligned.

### Equibiaxial extension

We next considered the equibiaxial extension problem to demonstrate the material anisotropy. We align fibers in the x-direction as before and apply an electric field in the fiber direction. The deformation gradient, left Cauchy Green tensor, unit fiber orientation, electric field, and fiber vector in the current configuration have components

$$ \begin{aligned} & {\mathbf{F}} = diag\left[ {\begin{array}{*{20}c} \lambda & \lambda & {\lambda^{ - 2} } \\ \end{array} } \right]\;\;{\mathbf{b}} = diag\left[ {\begin{array}{*{20}c} {\lambda^{2} } & {\lambda^{2} } & {\lambda^{ - 4} } \\ \end{array} } \right] \\ & {\mathbf{V}} = \left[ {\begin{array}{*{20}c} 1 & 0 & 0 \\ \end{array} } \right]^{T} \;\;{\mathbf{E}} = \left[ {\begin{array}{*{20}c} E & 0 & 0 \\ \end{array} } \right]^{T} \;\;{\mathbf{v}} = {\mathbf{FV}} = \left[ {\begin{array}{*{20}c} \lambda & 0 & 0 \\ \end{array} } \right]^{T} . \\ \end{aligned} $$

The stress expressions in the x- and y-direction are obtained from Eq. ( 20) and using ${\sigma }_{33}=0$ with the same nondimensional variables used prior, we have

40 $${\tilde{\sigma }}_{11}=\left({\lambda }^{2}-\frac{1}{{\lambda }^{4}}\right)+2{\overline{\mu }}_{feq}\left({\lambda }^{2}-1\right){\lambda }^{2}+2{\overline{\mu }}_{{fneq }}\left(\frac{{\lambda }^{2}}{{I}_{4}^{v}}-1\right)\frac{{\lambda }^{2}}{{I}_{4}^{v}}+\frac{{\overline{E} }^{2}}{{\lambda }^{2}},$$

41 $${\tilde{\sigma }}_{22}=\left({\lambda }^{2}-\frac{1}{{\lambda }^{4}}\right).$$

**Figure 7:**
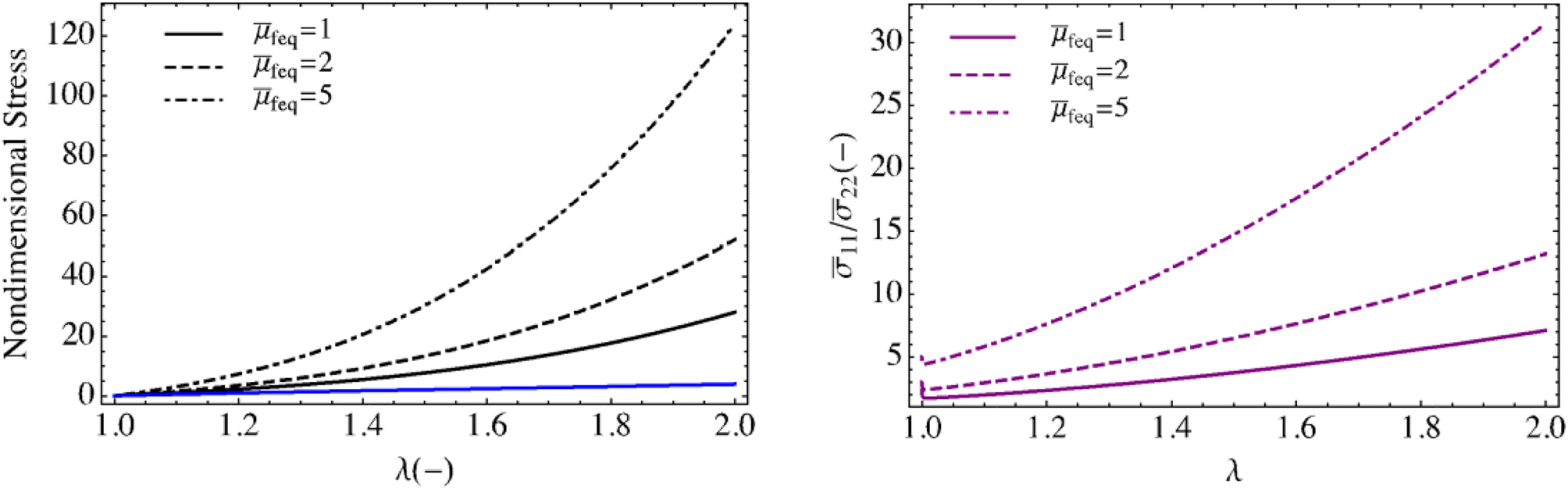
Passive nondimensional equibiaxial stress with varying nondimensional ${\overline{\mu }}_{feq}.$ (Black curves ${\tilde{\sigma }}_{11}$, blue ${\tilde{\sigma }}_{22}$, and magenta ${\tilde{\sigma }}_{11}/{\tilde{\sigma }}_{22}$).

Figure 7 shows the passive stress stretch relationships for various modulus ratios ${\overline{\mu }}_{feq}$. The stress ratio indicates the degree of mechanical anisotropy.

**Figure 8:**
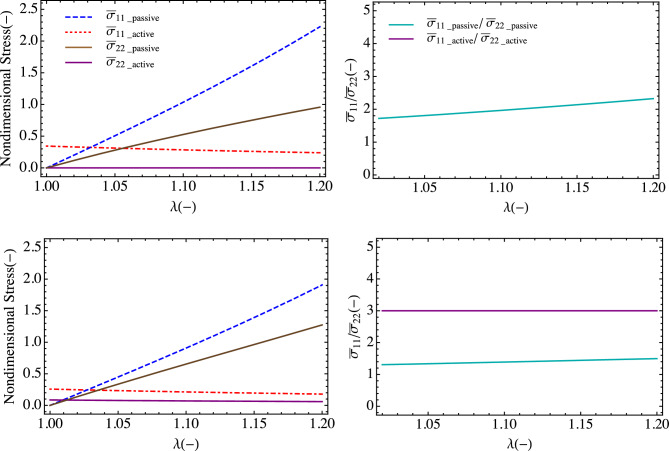
(left) Nondimensional total, active, and passive stresses in x- and y-directions for fiber orientations: (top) 0°, and (bottom) 30°. (right) Stress ratio as a function of stretch highlighting the nonlinear material anisotropy.

For a nonzero electric field in the fiber direction (12 MV/m), we calculate the total, active, and passive stress under equibiaxial isometric conditions for fibers oriented in the 11-direction 0° and for 30° (Fig. 8). As with the uniaxial case, the active part of the stress creates an initial offset to the stress, and with increasing stretch the passive term dominates. The plot of the stress ratios present an interesting view, which highlights the dominance of the active term especially for the initial stretch regime. This drop rapidly with increasing stretch. The passive anisotropy linearly increases with increasing stretch. The active term is relatively constant over the stretch.

## Concluding remarks

Inspired by soft fibrous active materials in biology and engineering, we developed constitutive model formulations to describe the activation of a soft fibrous material containing contractile elements. The model itself, summarized in Table 1, is constructed within a nonlinear continuum mechanics framework for electromechanically coupled inelastic materials undergoing finite deformations.

**Table 1:** Summary of final model equations.

Strain energy function:
$$\begin{aligned} {\Psi } & = \underbrace {{\frac{\mu }{2}\left( {I_{1} - 3} \right) + \frac{{\mu_{{{feq }}} }}{2}\left( {I_{4} - 1} \right)^{2} + \frac{{\mu_{{fneq }} }}{2}\left( {\frac{{I_{4} }}{{I_{4}^{v} }} - 1} \right)^{2} }}_{mechanical } - \underbrace {{\frac{{\varepsilon_{0} \varepsilon_{rf} }}{2}I_{11}^{f} }}_{electrical\text{-}fiber } \\ & \quad - \underbrace {{ \frac{{\varepsilon_{0} \varepsilon_{rm} }}{2}I_{11}^{m} }}_{electrical\text{-}matrix }, \\ \end{aligned}$$
Cauchy stress:
$$\begin{aligned} {\varvec{\sigma}} & = \underbrace {{\mu {\mathbf{b}} + 2\mu_{feq } \left( {I_{4} - 1} \right){\mathbf{v}} \otimes {\mathbf{v}} + 2\mu_{fneq } \left( {\frac{{I_{4} }}{{I_{4}^{v} }} - 1} \right)\frac{1}{{I_{4}^{v} }}{\mathbf{v}} \otimes {\mathbf{v}}}}_{mechanical } + \underbrace {{\varepsilon_{0} \varepsilon_{rf} \frac{{E_{f}^{2} }}{{I_{4} }}\frac{{\mathbf{v}}}{{\sqrt {I_{4} } }} \otimes \frac{{\mathbf{v}}}{{\sqrt {I_{4} } }}}}_{electrical\text{-}fiber } \\ & \quad + \underbrace {{\varepsilon_{0} \varepsilon_{rm} {\mathbf{e}}_{m} \otimes {\mathbf{e}}_{m} - \frac{{\varepsilon_{0} \varepsilon_{rm} }}{2}{\mathbf{e}}_{m} \cdot {\mathbf{e}}_{m} {\mathbf{I}}}}_{electrical\text{-}matrix } + p{\mathbf{I}}. \\ \end{aligned}$$
Evolution equation:
$$-{\tau }_{{neq }}=\eta {\dot{I}}_{4}^{v}$$
$${\tau }_{{neq }}=4{\mu }_{{fneq }}\left(\frac{{I}_{4}}{{I}_{4}^{v}}-1\right)\frac{{I}_{4}}{{I}_{4}^{v}}$$

The kinematics draw on those used in the mechanics community to model soft biological tissues. We consider this similarity a major advantage in light of new engineered living materials being developed. Here, the constitutive equations are particularized assuming a quadratic dependence on the electric field (the activation), reminiscent of the electrostatic coupling in dielectric elastomers, and which is in addition to the electrostatic coupling of the matrix. The coupled constitutive formulation highlights a new electromechanical coupling term to capture the field-based response of the contractile units. This intrinsic (or active) anisotropy alters the constitutive response by adding an initial offset (nearly constant) to the total stress. Because of orientation effects, the constitutive response can be amplified or muted depending on the electric field or fiber direction. The constitutive model could be further particularized to capture material phenomena of increasing complexity, for example, due to fiber kinematics, statistical fiber distributions, and number of fiber families. Computational implementation of the model in^62^ shows a method for carrying out numerical simulations of three-dimensional deformations of soft intrinsically anisotropic materials with various architectures. The work could be further extended to study other problems involving the universal deformations such as finite torsion and extension of circular bars, and inflation/inversion of spherical shells^63^.

## Data Availability

All data and models used in this study can be provided by the contact author (N.C. Goulbourne) upon request.
